# Topical Application of a Collagen Mimetic Peptide Restores Peripapillary Scleral Stiffness Reduced by Ocular Stress

**DOI:** 10.3390/ph18060875

**Published:** 2025-06-12

**Authors:** Lauren K. Wareham, Ghazi O. Bou Ghanem, Kristin L. Clark, Eric Schlumpf, Brian J. Del Buono, David J. Calkins

**Affiliations:** 1 Vanderbilt Eye Institute, Department of Ophthalmology and Visual Sciences, Vanderbilt University Medical Center, Nashville, TN 37232, USA; lauren.wareham@vumc.org (L.K.W.); ghazi.b.ghanem@vumc.org (G.O.B.G.); kristin.clark@vumc.org (K.L.C.); 2 Stuart Therapeutics, Inc., Stuart, FL 34994, USA; eric@stuarttherapeutics.com (E.S.); brian@stuarttherapeutics.com (B.J.D.B.)

**Keywords:** collagen, myopia, glaucoma, mimetic peptides, atomic force microscopy

## Abstract

**Background:** The biomechanical properties of ocular tissues are critical to physiological processes that span ocular development, aging, and disease. The structural integrity of these tissues is important in mediating how the eye responds to strain and stress that pose challenges to physiological homeostasis. In the posterior segment, the peripapillary sclera and lamina separate the intraocular chamber and the fluid-filled subarachnoid space. Degradation of each contribute to pathogenic progression in multiple conditions and are largely determined by the integrity and architecture of collagen fibers, especially type I collagen. **Methods:** We used atomic force microscopy to measure how stress induced by elevations in intraocular pressure impacts stiffness of the peripapillary sclera and glial lamina in the rat eye and whether changes in stiffness could be influenced by topical treatment of a reparative mimetic of type I collagen. **Results:** Four weeks of elevated intraocular pressure reduced Young’s modulus in peripapillary sclera and glial lamina, coincident with reduced anterograde transport along the optic projection to the brain. Reduction in tissue stiffness correlated with an increase in fragmented collagen. Topical application of collagen mimetic peptide during the period of elevation countered both. **Conclusions:** Collagen remodeling occurs in many ocular conditions that influence the peripapillary sclera and glial lamina, including glaucoma and myopia. Our results suggest that topical application of collagen mimetic peptides that intercalate with and repair collagen damaged by disease processes could serve to mitigate changes in tissue stiffness and integrity due to degraded collagen.

## 1. Introduction

The biomechanical properties of tissues of the eye are critical to a variety of physiological processes including development and aging and, importantly, response to disease-related stress and strain that can differ across populations [1,2]. The response to tissue stress can help predict the susceptibility of an individual to pathogenic processes and how likely tissues spanning the cornea to the optic nerve will recover structural homeostasis [3]. Tissue integrity, compliance, deformation, and recovery are critical determinants of conditions affecting the cornea, sclera, retina, and optic nerve head, including keratoconus, myopia, age-related macular degeneration, and glaucoma [4,5,6,7,8]. In some cases, tissue elasticity and reduced resilience conflate with aging and other risk factors to accelerate pathogenic progression [9]. Finally, the eye itself is constantly under strain from rotational and translational motion due to eye movements that are thought to contribute to chronic and progressive distortion of the sclera in axial myopia and to optic nerve head damage in glaucoma [10].

Tissue stiffness is the property that reflects its ability to resist or accommodate mechanical challenges, such as deformation or stretch that accompany changes in pressure. In general, stiffer tissue deforms less than compliant tissue, and changes in stiffness in one part of the eye can have profound effects on the response to stress of another. For example, stiffening of the peripapillary sclera can increase the capacity of the lamina cribrosa of the optic nerve head to resist biomechanical strain due to ocular pressure [11]. The mechanical properties of the sclera are critical determinants of eye shape, malformation of which can cause diminished visual acuity [12]. Similarly, scleral thinning and diminished scleral stiffness can lead to defects in the choroid and retina that are generally associated with the development of myopia [13,14]. Finally, stiffness of neural tissue, such as that found in the retina and optic nerve head, is an important factor in modulating local inflammation, glial migration, and scar formation, and the capacity for neuronal axons to grow and infiltrate central brain targets [15].

Scleral and optic nerve head biomechanics are significantly influenced by collagen architecture [16,17,18,19]. At the nerve head, collagen-rich tissue of the peripapillary sclera (PPS) surrounding the scleral canal adjoins the lamina cribrosa, a collagenous structure in primates and certain other mammals. The cribrosa is critical to supporting retinal ganglion cell axons as they exit the globe to form the optic nerve [20]. While rodents lack a lamina cribrosa, the analogous glial lamina of the optic nerve head contains honeycomb-like structures formed by collagen fibrils among a matrix of collagen-depositing astrocyte glia [21,22]. The specific architecture and orientation of the collagen network in the PPS-nerve head complex is both challenged by and determines the mechanical response to ocular stress [23].

Since the nerve head is important for providing the optic nerve structural support, changes in the biomechanical or structural integrity to collagen within the PPS and lamina may lead to tissue deformation and susceptibility to disease-related damage. For example, stress related to intraocular pressure (IOP) drives extensive tissue remodeling, including collagen breakdown by matrix metalloproteases (MMPs) like MMP-1 [24,25,26]. This can be accompanied by decreased collagen fibril density in human and non-human primate PPS and lamina [27]. Collagen remodeling and replacement observed in human, non-human primate, and rodent glaucomatous tissue may be a biological response to weakening and the resultant increased compliance of the nerve head in an attempt to restore biomechanical stability [28,29,30]. Thus, the pathophysiological response to ocular stress involves complex remodeling processes that affect the structural and biomechanical properties of the tissue [31]. Collagen degradation by MMPs in response to IOP in ocular tissues not only alters tissue biomechanics but also triggers a host of downstream pathogenic responses [32,33].

A recent study using atomic force microscopy (AFM) in rats showed that increased IOP decreased RGC axon and glial lamina (GL) tissue stiffness [34]. Here, we used AFM to investigate how elevations in IOP induced by microbead occlusion of the anterior chamber in the rat eye influence stiffness of the PPS and GL as assessed by Young’s modulus. We chose four weeks of elevations since in this model, this period of stress results in degradation of functional anterograde axonal transport along the optic projection to the brain, which is an early marker of degeneration [35,36,37]. Short, single-stranded collagen mimetic peptides (CMPs) have demonstrable capacity to anneal with high affinity preferentially to damaged collagen vs. intact collagen [38,39]. Previous work has shown that by doing so, CMPs restore the stiffness of ocular tissue degraded by MMP activity ex vivo [40]. Thus, we next determined whether IOP-induced changes in tissue stiffness could be altered by topical application of a CMP. In line with previous work [34], we found that IOP stress significantly decreased the stiffness of both the PPS and GL, coincident with elevated levels of fragmented collagen compared to control eyes. Finally, topical application of CMP during the period of elevation counters the IOP-induced reduction in tissue stiffness of the PPS and GL compared to vehicle. This outcome correlated with changes in collagen deposition and fragmentation, which may have been impacted by CMP application. Together our findings suggest that topical CMP application restores stiffness of tissues in the posterior challenged by stress due to elevations in IOP.

## 2. Results

### 2.1. IOP Elevation Decreases Young’s Modulus for Peripapillary Sclera and Glial Lamina

**Figure 1:**
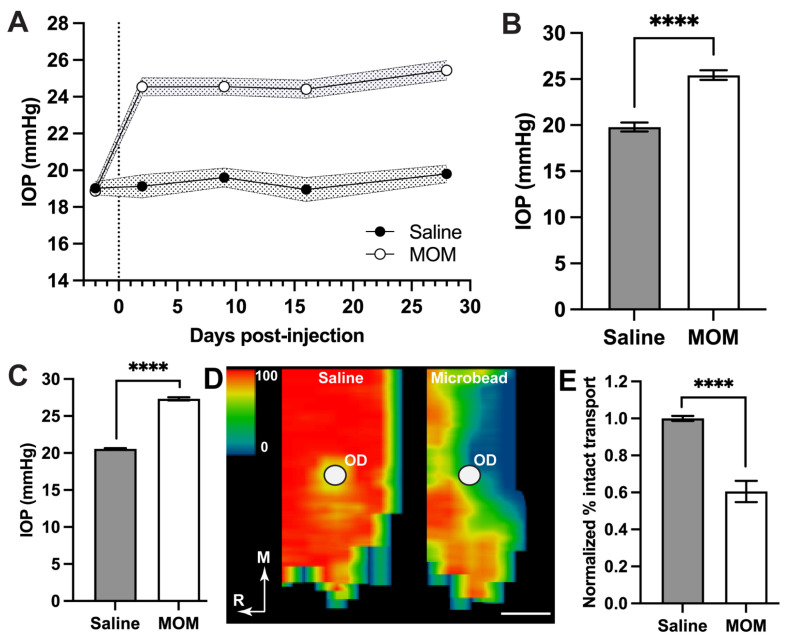
Intraocular pressure (IOP) and early axonopathy. (**A**) Mean IOP following bilateral saline or microbead occlusion (MOM) on day 0 (dotted line); standard error shown by shaded region. Day -2 represents baseline IOP. (**B**) Mean IOP during 4-week period differs between saline vs. microbead eyes (unpaired *t*-test with Welch’s correction; **** *p* < 0.0001; means ± SEM). (**C**) Historical IOP averaged between independent rat cohorts following 4 weeks of microbead-induced elevation (27.3 ± 0.2 mmHg, n = 14 eyes) vs. saline-injected control eyes (20.6 ± 0.41 mmHg, n = 12); unpaired *t*-test with Welch’s correction; **** *p*< 0.0001). (**D**) Representative retinotopic maps reconstructed from serial sections through superior colliculus showing intact transport of cholera toxin B from a saline vs. microbead eye following 4 weeks of elevated IOP. Density of the transported CTB signal ranges from 0% (blue) to 50% (green) to 100% (red). Representation of the optic disk in the retina (OD) lacks transport due to absence of retinal ganglion cells from that region. Medial (M) and rostral (R) orientations are indicated; scale = 500 μm. (**E**) Percentage of intact anterograde CTB transport to the superior colliculus from saline (n = 12) vs. microbead (n = 14) eyes following 4 weeks of elevated IOP (as shown in **C**) normalized to the average of saline (unpaired *t*-test with Welch’s correction; **** *p*< 0.0001; data presented as means ± SEM.

Baseline IOP measurements were taken for several days prior to bilateral microbead occlusion or saline injection (Figure 1). Microbead occlusion elevated IOP by an average of 29% in microbead eyes compared to saline over the 4-week experimental period (19.80 ± 0.48 vs. 25.44 ± 0.53 mmHg; Figure 1A,B; *p* < 0.0001). This elevation is similar to our historical IOP measurements published from rats for the same 4-week period (Figure 1C), which we have shown results in a 40% reduction in anterograde transport of cholera toxin B (CTB) from the retina to the superior colliculus along the optic nerve (Figure 1D,E; [35,36]). Degradation of active transport of CTB is an early event marking optic nerve degeneration in models of glaucoma, preceding frank degeneration of both retinal ganglion cells and their axons [37,41]. Thus, our choice of a 4-week period of IOP elevation is relevant to the progression of neurodegeneration in this model of glaucoma.

**Figure 2:**
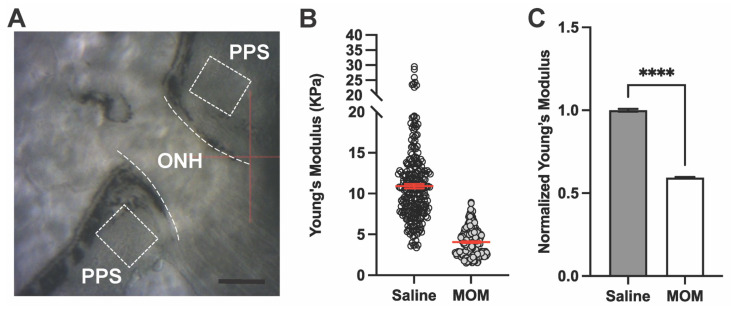
IOP elevation decreases Young’s modulus of the peripapillary sclera (PPS). (**A**) AFM microscope view showing optic nerve head (ONH; dashed lines) and PPS (dashed boxes) regions of interest. Scale bar = 200 μM. (**B**) Representative Young’s modulus values from one PPS location for each condition in one animal, saline vs. MOM (n = 256 data points per condition). Red lines indicate data means ± SEM. (**C**) Mean Young’s modulus from PPS across all biological repeats normalized with respect to saline eye measurements (unpaired *t*-test with Welch’s correction; ****, *p* < 0.0001). Data are presented as means ± SEM.

Next, we used AFM to measure stiffness as determined by Young’s modulus of the PPS and GL in fresh-frozen tissue sections from the eyes subjected to elevated IOP shown above (Figure 1A,B). AFM force curves for PPS were taken at locations ranging 300–500 μm from the optic nerve edge (dashed ROI; Figure 2A). Measurements of Young’s modulus for the PPS from one representative saline- and one microbead-injected eye are shown in Figure 2B, demonstrating a decrease after 4 weeks of IOP elevation. When averaged across our sample of eyes (Figure 2C), Young’s modulus of the PPS in microbead eyes decreased by 40.6% compared to saline control eyes (10.91 ± 0.27 vs. 4.05 ± 0.10 KPa, respectively; *p* < 0.0001), indicating decreased stiffness of the tissue after IOP insult.

**Figure 3:**
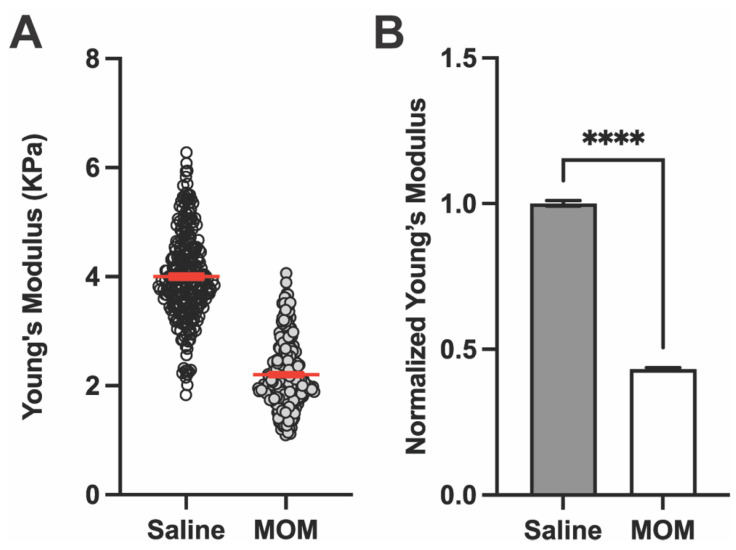
IOP elevation decreases Young’s modulus of the glial lamina. (**A**) Representative Young’s modulus values from one GL location for a saline vs. MOM animal (n = 256 data points each). Red lines indicate mean ± SEM. (**B**) Young’s modulus from GL across all biological repeats normalized with respect to saline eye measurements (Unpaired *t*-test with Welch’s correction; **** *p* < 0.0001). Data presented as means ± SEM.

Representative AFM measurements of Young’s modulus for the GL in a saline and microbead eye are shown in Figure 3A, which also showed a decrease after 4 weeks of IOP elevation. Again, when averaged across the complete set of samples (Figure 3B), Young’s modulus of the GL decreased by 56.7% with IOP elevation compared to control (4.00 ± 0.05 vs. 2.20 ± 0.04 KPa, respectively; *p* < 0.0001), indicating decreased stiffness of the tissue.

### 2.2. Collagen Mimetic Peptides Counter the Influence of IOP Elevation on Tissue Stiffness

**Figure 4:**
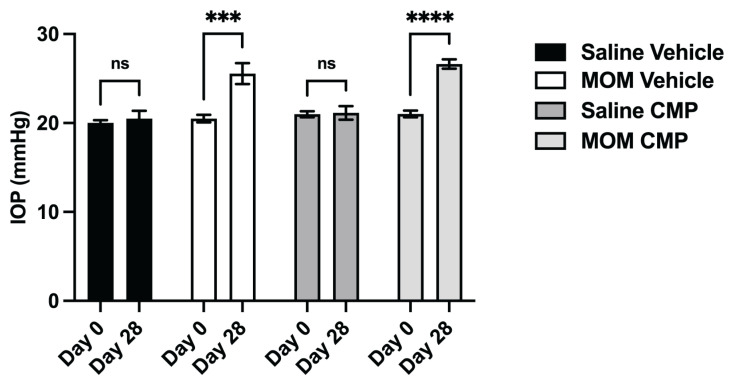
Intraocular pressure (IOP) elevation in rats with microbead injection and topical vehicle or CMP. Mean IOP at baseline (Day 0) and 4 weeks following bilateral saline or microbead injection with either topical vehicle or CMP. Animal numbers as described in Methods. Saline-injected eyes with vehicle or CMP treatment did not exhibit IOP elevation (ns; *p* = 0.640 and *p* = 0.900, respectively). Microbead-injected eyes with vehicle treatment showed elevated IOP compared to baseline (***, *p* = 0.0002), as did those with CMP treatment (****, *p* < 0.0001). IOP at week 4 did not differ between MOM vehicle and CMP groups (*p* = 0.440). Data are presented as means ± SEM. (2-way ANOVA).

We recently demonstrated the capacity of CMPs to restore the stiffness of MMP-degraded tissue in the GL and PPS in ex vivo tissue [40]. Next, we explored whether topically applied CMP would have a similar effect in vivo. In additional rat cohorts, IOP was elevated bilaterally via microbead injection for comparison with bilateral saline controls. One week following injection, vehicle (1X PBS) or CMP (200 μM) was applied once daily as a topical drop (20 μL). As expected, microbead-induced IOP elevation did not differ between vehicle- and CMP-treated eyes (24.7% and 26.7% for 4 weeks, respectively, Figure 4, *p* = 0.440), though each differed compared to the corresponding saline group (*p* = 0.0002; *p* < 0.0001, respectively); IOP for saline-injected eyes in either treatment group did not differ over the 4-week period compared to baseline (*p* = 0.640 and 0.900, respectively). Thus, topical application of CMP had no influence on IOP, similar to results following intravitreal injection [42,43].

**Figure 5:**
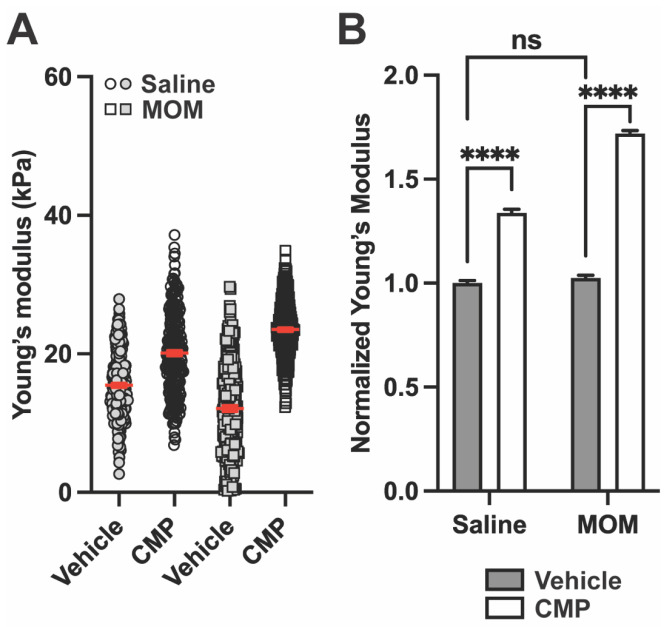
CMP increases PPS Young’s modulus after saline and microbead injection. (**A**) Representative Young’s modulus for PPS from one section each from saline and MOM eyes receiving either vehicle or CMP (for vehicle, n = 2560 measurements for saline and 768 for MOM; for CMP, n = 2560 measurements for saline and 5120 for MOM). In the vehicle group, MOM decreased Young’s modulus vs. the saline eye. In the CMP group, treatment increased Young’s modulus for both saline and MOM eyes compared to vehicle, though the effect was greater in MOM PPS vs. saline. Red lines indicate mean ± SEM. (**B**) Young’s modulus normalized to vehicle-treated saline eyes from all biological repeats (One-way ANOVA; ****, *p* < 0.0001). Data are presented as means ± SEM.

Representative Young’s modulus measurements for the PPS are shown in Figure 5A. CMP treatment increased the stiffness of the PPS for both saline- and microbead-injected eyes. In saline-injected animals, tissue stiffness increased in CMP-treated eyes compared to the vehicle (Figure 5A). Similarly, in microbead-injected eyes, CMP increased the stiffness of the PPS (Figure 5A). When all biological repeats are pooled and normalized to saline-injected vehicle, CMP increased PPS stiffness by 28.90% in saline eyes and by 52.90% microbead-injected eyes (*p* < 0.0001; Figure 5B). Interestingly, PPS stiffness was similar in vehicle-treated saline and microbead eyes (*p* = 0.324).

**Figure 6:**
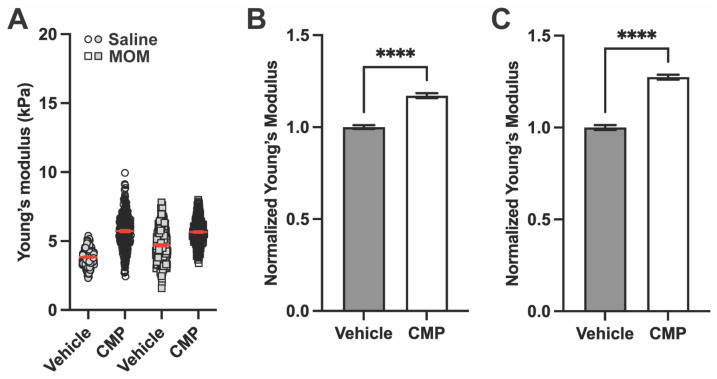
CMP increases stiffness of the GL after saline and microbead injection. (**A**) Representative Young’s modulus values from one section for each group (n = 1536 measurements for saline vehicle, 1526 for saline CMP, 768 for MOM vehicle, and 3072 for MOM CMP). In the CMP group, treatment increased Young’s modulus for both saline and MOM eyes compared to vehicle. Red lines indicate mean ± SEM. Young’s modulus data normalized to saline vehicle control from all biological repeats in (**B**) saline-injected and (**C**) microbead-injected eyes (One-way ANOVA; **** *p* < 0.0001). Data are presented as means ± SEM.

Representative Young’s modulus measurements for the GL are shown in Figure 6A. In saline-injected eyes, CMP increased tissue stiffness compared to vehicle. In microbead-injected eyes, CMP also increased GL stiffness compared to vehicle (Figure 6A). When all biological repeats are pooled and normalized to data from vehicle-treated saline eyes, CMP increased GL stiffness by 15.67% in saline eyes and by 33.33% in microbead eyes (*p* < 0.0001; Figure 6B,C). We also observed a decrease in stiffness between saline and microbead eyes in the vehicle group as expected based on measurements from untreated tissue (see Figure 3).

### 2.3. Collagen Mimetic Peptides Reduce Levels of Fragmented Collagen

**Figure 7:**
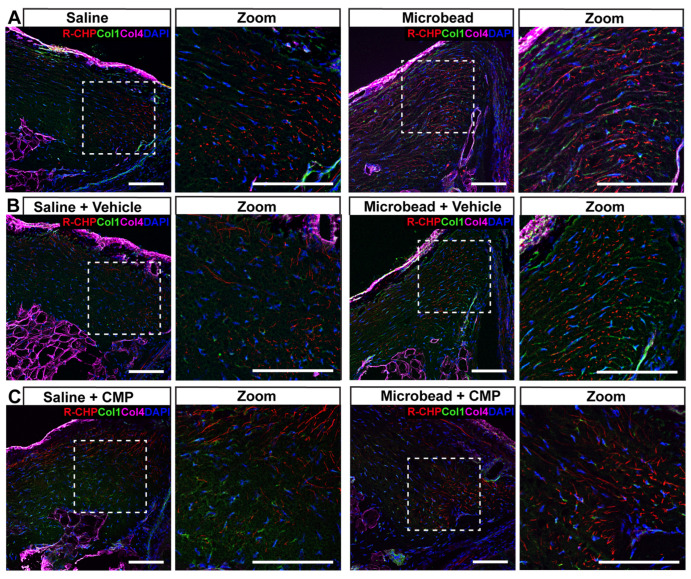
Fragmented collagen in peripapillary sclera. Representative confocal micrographs of PPS from a (**A**) saline (left) and microbead (right) eye without treatment labeled with antibodies to collagen I (green) and collagen IV (magenta) together with R-CHP (red) to detect fragmented collagen. Cell nuclei shown by DAPI staining (blue). Dashed boxes represent zoomed regions of interest. (**B**) In vehicle-treated PPS from microbead eyes, collagen I and R-CHP tend to increase, while CMP treatment (**C**) tends to reduce the area labeled by R-CHP. Scale bars = 100 μm.

The AFM measurements presented indicate that elevation of IOP decreases Young’s modulus for both PPS and GL and that topically applied CMP counters this decrease. To assess whether collagen content or integrity in either tissue could influence our measurements, we applied a hybridizing peptide highly specific for protease-degraded collagen with negligible binding to intact collagen in sections from the same eyes imaged with AFM (R-CHP; [40,44,45,46]). In PPS from saline-injected eyes without any treatment, R-CHP staining was evident within the sclera, while immune-labeling for collagen IV highlighted the scleral wall and other connective tissues (Figure 7A, left). For PPS subjected to microbead-induced elevations in IOP, R-CHP increased along with immuno-labeling against collagen I (Figure 7A, right). This pattern was similar for PPS from saline- and microbead-injected eyes with vehicle treatment (Figure 7B). For CMP-treated PPS, R-CHP was less diffuse, and any changes in collagen I were less apparent (Figure 7C). As the negative control, microbead-injected tissue, in which the primary antibodies for collagens I and IV were omitted, show no label.

**Figure 8:**
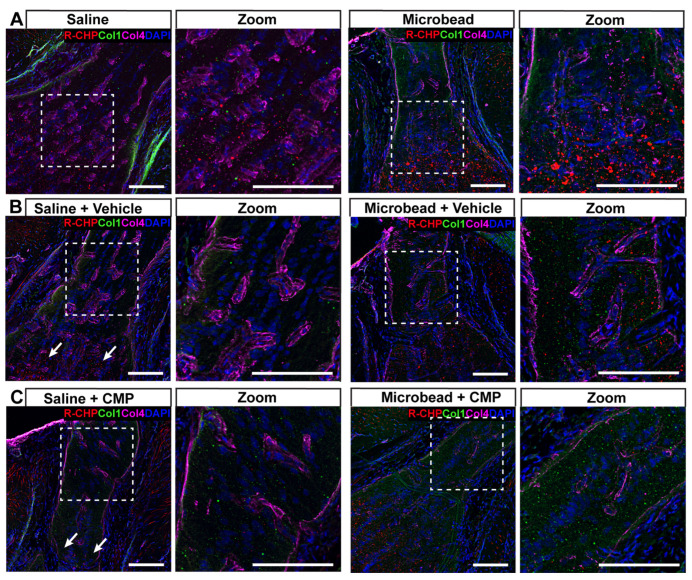
Fragmented collagen in glial lamina. Representative confocal micrographs of GL from a (**A**) saline (left) and microbead (right) eye without treatment labeled with antibodies against collagen I (green) and collagen IV (magenta) together with R-CHP (red) to detect fragmented collagen. Cell nuclei shown by DAPI staining (blue). Dashed boxes represent zoomed regions of interest. (**B**) In vehicle-treated GL from microbead eyes, collagen I and R-CHP tend to increase, while CMP treatment (**C**) tends to reduce the area labeled by R-CHP while increasing collagen I relative to saline-eye GL. Scale bars = 100 μm.

In the naïve GL (Figure 8A), microbead-induced elevations in IOP increased R-CHP especially near the posterior region where axonal myelination occurs, while collagen IV was readily detected in both saline and microbead tissue. Interestingly, as with the PPS, vehicle-treated GL from microbead eyes also showed increased collagen I compared to saline eyes (Figure 8B). In this case, treatment with CMP again appeared to reduce fragmented collagen labeled by R-CHP but also increased collagen I in microbead GL compared to saline (Figure 8C).

## 3. Discussion

In our previous work, we demonstrated the potential of CMPs to restore ocular tissue resilience in ex vivo eye tissue sections. After ex vivo MMP-induced degradation of collagen, we showed that CMP applied to the tissue increases Young’s modulus using AFM [40]. Here, we explored the effect of stress induced by IOP on Young’s modulus of posterior segment tissues once again using AFM at a key point in disease progression (four weeks of IOP elevation) where axonopathy is apparent in the microbead model (Figure 1). We found that IOP elevation reduced stiffness in the PPS and the GL. This is in line with recent work that used AFM in a similar way to evaluate tissue stiffness of the GL and RGC axons after elevated IOP [34]. Specifically, in our work, an IOP elevation of 29% over a period of a month was sufficient to reduce the stiffness of PPS by nearly 41% and that of GL by nearly 57% (Figure 2 and Figure 3). This reduction is consistent with our earlier finding from ex vivo ocular sections that the GL is far more susceptible to MMP-1 induced degradation and, therefore, to reduced stiffness than is the PPS [40].

In additional cohorts of rats with bilateral elevations in IOP of similar magnitude (Figure 4), we assessed whether the observed decrease in Young’s modulus could be countered by topical application of CMP. Application of CMP during the period of elevation increased Young’s modulus of the PPS by 41% compared to vehicle (Figure 5), while the increase for the GL was 18% (Figure 6). This is not unexpected, since previous work in the microbead model demonstrated the capacity for CMP to reach the posterior tissues of the eye [43]. Interestingly, CMP also increased Young’s modulus for both PPS and GL from saline-injected (normal IOP) eyes. The specificity of the class of CMPs tested here for annealing to and repairing damaged collagen triple helices is well-established [39]. Given this specificity, the increased Young’s modulus in tissue from control, saline-injected eyes could indicate a degree of collagen remodeling in the posterior segment due to the injection itself. In other experimental models, there is strong precedent for perturbations of the anterior eye causing downstream remodeling in the posterior segment [47], which is linked to collagen homeostasis and repair [33].

We hypothesize that our AFM results may reflect the influence of CMP on levels of fragmented collagen. More extensive longitudinal studies for different periods of elevation are needed to quantitatively assess this possibility. However, there are signs that increased tissued stiffness as measured by Young’s modulus correlate with changes in levels of fragmented collagen as assessed by R-CHP, which is highly specific for damaged collagen because of its neutral and hydrophilic nature [44,48]. Previously we have shown that in ex vivo eye sections, treatment with MMP increased R-CHP levels in both PPS and GL, an effect that was countered effectively with CMP applied to the sections [40]. Similarly, microbead-induced elevations in IOP appeared to increase R-CHP in both PPS (Figure 7) and GL (Figure 8); topical CMP appeared once again to counter this increase. Interestingly, microbead elevations in IOP also increased levels of collagen I in the PPS; CMP prevented this influence. In the GL, CMP had the opposing effect, increasing intact collagen I in the GL. While the increases in Young’s modulus we observed with CMP treatment were highly significant (Figure 5 and Figure 6), how these increases reflect remodeling of collagen in PPS and GL is apparently more complicated. Whether the CMP increases stiffness by intercalating into damaged collagen, or whether CMP is triggering remodeling in tissues remains to be resolved. Future studies to elucidate the exact mechanism of CMP action in tissues in vivo are, therefore, warranted.

The stiffness of ocular tissues critical in maintaining compliance, including the sclera, is predominantly determined by collagen, which together with other ECM proteins form a dynamic landscape for biomolecular signaling. Tissue stiffness, along with deformation and recovery, can impact the progression of diseases affecting the cornea, sclera, retina, and optic nerve head, including keratoconus, myopia, age-related macular degeneration, and glaucoma [4,5,6,7,8]. The structural and biomechanical properties of the sclera and lamina determine to what extent IOP-related stress can cause deformation and architectural degradation of the optic nerve head [31,49]. Furthermore, scleral resistance to stress influences IOP-dependent lamina strain [50].

In glaucoma, scleral integrity can be differentially affected; stiffness decreases in non-human primates early in progression and with minimal IOP elevation, whereas stiffness increases with more chronic, or greater IOP elevations [51,52]. Collagen fibril alignment is also altered in human glaucomatous eyes [53,54]. Alignment of fibrils affects lamina cribrosa strain; moderate anisotropy offers more protection against strain than isotropy or high anisotropy [18]. This change may be in part due to expression of MMPs in posterior tissues that breakdown collagen triggering structural reorganization, and thus, biomechanics [55,56]. Various approaches to cross-link proteins, including the use of glutaraldehyde, counter collagenase degradation of scleral tissue to reduce strain and deformation, highlighting the potential benefit of increased stiffness [11], particularly as applicable to myopia [57]. However, the therapeutic potential of targeting tissue stiffness at the molecular level in glaucoma is not clearly established. Some findings support the use of scleral matrix stiffening through collagen crosslinking as a potential avenue to decrease IOP-induced strain at the optic nerve head to provide neuroprotection in glaucoma [58]. However, in mice exposed to chronic levels of elevated pressure gross scleral ECM reorganization increases overall tissue stiffness [59]; prevention of ECM remodeling in the same model decreases RGC degeneration [60]. Targeted scleral stiffening does not impact cell survival in murine models of glaucoma [61,62], and mice lacking lysyl oxidase-1 exhibit loss of vision independent of IOP elevation along with increased scleral stiffness [63]. Thus, targeting tissue biomechanics alone as a therapeutic strategy in glaucoma requires further exploration and understanding.

Our results suggest that CMP applied topically to the ocular surface has the capacity to increase the stiffness of tissues in the posterior segment. Importantly, CMPs do not cross-link collagen, but rather intercalate into MMP-degraded triple helices to restore their structure and strength [38,39], they also appear to help realign collagen upon repair following MMP-induced damage [64]. Changes to the native structure of collagen impact its biomechanical properties, including its resilience to deformation [33]. An in vitro study exploring the impact of MMP degradation on Young’s modulus of individual collagen I fibers saw a 34% decrease in stiffness of the fibers after degradation [65]. Furthermore, degrading collagen in ex vivo corneal samples decreased stiffness of both the tissue and purified collagen fibrils [66]. There are some limitations of the current study to consider, for example, the freeze–thaw and cryosection procedure may influence the mechanical properties of the tissue itself. However, freeze-thawing porcine sclera does not cause significant changes in mechanical properties of the tissue as detected by AFM [67]. This is in contrast to other soft tissues including the human tendon [68] and lens [69] where freeze–thaw cycles have been demonstrated to change the inherent biomechanical properties of the tissue. Although more investigation is needed to determine the effect of freeze-thawing on mouse ocular tissue, our conclusions are drawn from relative comparisons between groups of tissue that has been treated in an identical manner. In fact, many studies have implemented this same methodology to measure the stiffness of a variety of ocular structures including the mouse trabecular meshwork [70], optic nerve [71], porcine cornea [72], rat glial lamina and RGC axons [73], and human lamina cribrosa and sclera [74]. Our study assesses stiffness in microstructures of the PPS and GL, which also includes collagen, elastin, and cellular structures, rather than evaluating strain and stress relationships in whole tissue. Follow up studies that assess the effect of CMPs on radial and circumferential strain in these tissues without disrupting tissue structure will help to evaluate the therapeutic potential of CMPs. Finally, our study investigated the effect of CMP dosed once per day for a period of 4 weeks post elevation of IOP. In future studies, the effect of repeated dosing of CMP over longer periods of time is warranted to evaluate CMP efficacy. The CMP used in this study was the same mimetic peptide used in a recent phase 2 clinical trial where the CMP was applied topically twice daily in humans over a period of 4 weeks for the treatment of dry eye disease [75]. In that clinical trial, the topical application of the peptide was well-tolerated in reducing symptoms of the disease [75]. Future studies should evaluate tolerance and immune response to the CMP in the treatment of glaucoma. In addition, trials to evaluate ocular penetration after topical application of the mimetic are currently ongoing. The results of the trial will further strengthen the premise for the use of CMPs as topical agents for the treatment of posterior segment tissues.

Tissue biomechanics and ECM architecture not only impact the structural integrity of tissues but have the potential to alter local cellular signaling pathways as well. Our understanding of the extracellular landscape is evolving; the ECM is not just an inert biological scaffold, rather, the ECM is emerging as a biologically active signaling milieu [33]. Collagen in the ECM harbors multiple receptor binding sites for integral biological signaling pathways including the complement cascade [33]. Changes to collagen structure, such as its proteolysis in disease, can augment local signaling pathways, for example, MMP-driven collagen fragmentation uncovers binding domains for proteins such as integrins [33]. Here, we have demonstrated the potential of CMPs to repair IOP-induced decreases in tissue stiffness, presumably due to fragmented collagen. Since collagen damage in the eye may be evident early in conditions such as myopia and glaucoma, the use of CMPs to repair collagen damaged by tissue strain or stress may be an effective preventative or restorative intervention.

## 4. Materials and Methods

### 4.1. Animal Model

Male Brown Norway rats (n = 15) at 3 months of age were obtained from Charles River Laboratories (Wilmington, MA, USA). For AFM studies in microbead vs. saline, n = 6. We also used an additional cohort of n = 9 rats for topical vehicle vs. CMP treatment. The study adhered to the ARVO Statement for the Use of Animals in Ophthalmic and Vision Research and was approved by the Institutional Animal Care and Use Committee of Vanderbilt University Medical Center. Rats were housed in a facility managed by the Vanderbilt University Division of Animal Care with a 12 h light/dark cycle (lights on at 6:30 a.m. and off at 6:30 p.m.) and ad libitum access to water and standard rat chow. Intraocular pressure was measured in awake rats using the Tono-Pen XL rebound tonometer (Medtronic Solan, Jacksonville, FL, USA) prior to and following bilateral injection of 5 µL of 15 µm polystyrene microbeads (Invitrogen, Eugene, OR, USA) or an equivalent volume of saline into the anterior chamber of the eye, following our published protocol [76,77].

### 4.2. Collagen Mimetic Peptide

The particular CMP used is a 21-residue single-strand peptide consisting of a 7-repeat sequence of proline (Pro) and glycine (Gly) as (Pro-Pro-Gly)_7_, the same as that used in our previous study [40]. This class of CMP mimics the structure of collagen-binding peptides known for their ability to intercalate into damaged type I collagen and reform its triple helical structure [39]. It was synthesized using standard solid-phase peptide synthesis (SPPS) chemistry and obtained from Bachem, AG (Frechen, Germany). One week following saline or microbead injection, 20 μL of CMP at a final concentration of 200 μM in 1X PBS or an equivalent volume of 1X PBS (vehicle) was applied once daily as a drop to the ocular surface.

### 4.3. Cholera Toxin B Assessment of RGC Axon Transport in Brain

Two days before sacrifice, we anesthetized rats with 2.5% isoflurane and injected both eyes intravitreally with 1.5 μL of 1 mg/mL solution of cholera toxin subunit B (CTB) conjugated to Alexa-488 (Molecular Probes, Eugene, OR, USA) [34,35]. Two days later, the rats were perfused transcardially with 1X PBS followed by 4% paraformaldehyde, and dissected tissues were cryoprotected in 30% sucrose. We prepared coronal midbrain Sections (50 µm thick) on a freezing sliding microtome and photographed sections of the superior colliculus using a Nikon Ti Eclipse microscope (Nikon Instruments Inc., Melville, NY, USA). We quantified the intensity of the CTB signal (intact transport) within the collicular retinotopic map using a custom ImagePro macro (Media Cybernetics, Bethesda, MD, USA) [35,42].

### 4.4. Tissue Preparation

Following euthanasia of rats with isoflurane anesthesia and decapitation, orientation of the eye was indicated by making a mark on the sclera at the superior-nasal quadrant using a marker pen on both eyes. Eyes were rapidly enucleated and placed in ice-cold phosphate-buffered saline (1X PBS). The eyes were then bisected posterior to the equator, and the anterior segment and lens were rapidly removed. The posterior segment containing the optic nerve head and PPS was immediately embedded in Optimal Cutting Temperature medium (OCT, Fisher Healthcare, Waltham, MA, USA, reference #4585) for cryo-sectioning in the same orientation for each eye. Sagittal eye sections were cut at 20 μm thickness and mounted on Poly-D-Lysine (PDL; Cat no: A38904-01–ThermoFisher Scientific, Frederick, MD, USA) coated glass coverslips. Samples were stored on dry ice for same-day Atomic Force Microscopy (AFM) imaging. Note samples remained frozen until AFM analysis for no longer than 4 h.

### 4.5. Atomic Force Microscopy (AFM)

Tissue stiffness was measured using AFM as previously described [40]. Sections on coverslips were mounted onto the AFM equipment for visualization of the peripapillary sclera (PPS) and glial lamina (GL). Tissue stiffness was measured using PeakForce Quantitative Nanomechanical Mapping (QNM) in Fluid AFM imaging mode (Bruker, Santa Barbara, CA, USA). A SAA-SPH-5UM probe (Bruker, Santa Barbara, CA, USA) with a 5 μm end radius and a 0.25 N/m nominal spring constant was used to indent the PPS for stiffness measurements. A separate probe, CP-PNPL-SiO-D (Bruker, Santa Barbara, CA, USA), with a 5 μm tip radius and 0.08 N/m spring constant, was employed for GL stiffness measurements. AFM measurements were taken at the point in the force-indentation curve where the relationship between the applied force and the sample deformation was linear, allowing for the direct calculation of the tissue’s elastic modulus (Young’s modulus). Thus, force–displacement curves were analyzed using the Hertzian linearized model assuming a Poisson’s ratio of 0.5 with Bruker curve fitting software (NanoScope Analysis 1.5) to determine the elastic modulus (Young’s modulus). Before data collection, the probe was calibrated in liquid using the thermal tune method with the addition of 1X PBS [78]. In each tissue location, force volume maps were acquired using a scan size of 10 μm, with 16 samples per line for a total of 256 force–displacement curves, at a scan rate of 1 Hz. Up to 7 distinct PPS locations approximately 300–500 μm away from the edge of the optic nerve head were used for baseline or single measurements in each PPS sample, and up to 6 distinct locations per sample were chosen as baseline or single measurements in the GL. For each animal, at least three tissue sections were measured.

### 4.6. Immunohistochemistry

After AFM measurements, tissues were fixed in 4% paraformaldehyde (PFA) solution for 5 min and washed with 1X PBS at room temperature. Auto-fluorescence was quenched by adding 0.1% sodium borohydride/1X PBS for 30 min at room temperature. Tissue was washed 2× for 10 min per wash in 1x PBS solution. The tissue was then blocked in a solution containing 5% normal donkey serum (NDS; 017-000-121, Jackson ImmunoResearch Laboratories, Inc., West Grove, PA, USA) and 0.1% Triton X-100/1x PBS for 1 h at room temperature. After blocking, the tissue was placed in primary antibody solution (3% NDS/0.1% Triton X-100 in 1X PBS) with 20 µm collagen-hybridizing peptide (R-CHP, Cy3 Conjugate; 3Helix, Salt Lake City, UT, USA) and 1:100 anti-mouse collagen-1 (Ab6308, Abcam, Waltham, MA, USA) and anti-rabbit collagen IV (Ab6586, Abcam, Waltham, MA, USA). Samples were covered with paraffin overnight at 4 °C protected from light. The next day, the tissue was washed 3× for 10 min per wash in 1X PBS. Tissue was then placed in a secondary antibody solution (1% NDS/0.1% Triton X-100 in 1X PBS) containing 1:400 Donkey anti-mouse Alexa Fluor-488 (715-546-150, Jackson ImmunoResearch Laboratories, Inc., West Grove, PA, USA) for 2 h at room temperature, protected from light. The tissue was then washed 3× in 1X PBS for 10 min per wash and mounted in DAPI Fluoromount-G (0100-20, SouthernBiotech, Birmingham, AL, USA) for confocal imaging.

### 4.7. Confocal Microscopy

Fluorescent optic nerve head tissue images were taken using a Nikon Ti-E spinning disk confocal microscope and a 20× or 40× objective.

### 4.8. Statistical Analyses

All data are presented as mean ± standard error of the mean (SEM) unless otherwise specified. Graphs were generated, and statistical analyses were conducted using GraphPad Prism version 9.0 software (GraphPad Software, San Diego, CA, USA). The normality of the data was assessed using the Shapiro–Wilk test. If the data demonstrated a normal distribution, parametric statistical analyses such as *t*-tests and analysis of variance (ANOVA) were performed. For data that did not follow a normal distribution, non-parametric *t*-tests were employed, e.g., the Mann–Whitney test as detailed in the figure legends. Statistical significance was defined as a *p*-value of less than 0.05.

## Data Availability

The original contributions presented in the study are included in the article, further inquiries can be directed to the corresponding author.

## Abbreviations

The following abbreviations are used in this manuscript:

AFM	Atomic force microscopy
CTB	Cholera toxin B
CMP	Collagen mimetic peptide
ECM	Extracellular matrix
IOP	Intraocular pressure
GL	Glial lamina
MMP	Matrix metalloprotease
NDS	Normal donkey serum
ONH	Optic nerve head
PBS	Phosphate-buffered saline
PDL	Poly-D-Lysine
PPS	Peripapillary sclera
RGC	Retinal ganglion cell
